# Lowering the glycemic index of white bread using a white bean extract

**DOI:** 10.1186/1475-2891-8-52

**Published:** 2009-10-28

**Authors:** Jay K Udani, Betsy B Singh, Marilyn L Barrett, Harry G Preuss

**Affiliations:** 1 Medicus Research LLC, Northridge, CA 91325, USA; 2 UCLA School of Medicine, Department of Medicine, Los Angeles, CA 90024, USA; 3 Pharmacognosy Consulting, Mill Valley, CA 94941, USA; 4 Georgetown University Medical Center, Department of Physiology, Washington DC 20057, USA

## Abstract

**Background:**

Phase 2^® ^is a dietary supplement derived from the common white kidney bean (Phaseolus vulgaris). Phase 2 has been shown to inhibit alpha-amylase, the complex carbohydrate digesting enzyme, in vitro. The inhibition of alpha-amylase may result in the lowering of the effective Glycemic Index (GI) of certain foods. The objective of this study was to determine whether the addition of Phase 2 would lower the GI of a commercially available high glycemic food (white bread).

**Methods:**

An open-label 6-arm crossover study was conducted with 13 randomized subjects. Standardized GI testing was performed on white bread with and without the addition of Phase 2 in capsule and powder form, each in dosages of 1500 mg, 2000 mg, and 3000 mg. Statistical analysis was performed by one-way ANOVA of all seven treatment groups using unadjusted multiple comparisons (t tests) to the white bread control.

**Results:**

For the capsule formulation, the 1500 mg dose had no effect on the GI and the 2000 mg and 3000 mg capsule doses caused insignificant reductions in GI. For the powder, the 1500 mg and 2000 mg doses caused insignificant reductions in the GI, and the 3000 mg dose had a significant effect (-20.23 or 34.11%, p = 0.023)

**Conclusion:**

Phase 2 white bean extract appears to be a novel and potentially effective method for reducing the GI of existing foods without modifying their ingredient profile.

**Trial Registration:**

Trial Registration: ISRCTN50347345

## Background

The glycemic index (GI) describes the blood glucose response following consumption of a carbohydrate containing test food relative to a carbohydrate containing reference food, typically glucose or white bread. The GI was originally designed for people with diabetes as a guide to food selection, with the advice to select foods with a low GI. The benefits of low GI diets have been documented with epidemiological data. Low GI diets appear to decrease the risk of developing type II diabetes [1,2] and coronary heart disease [3]. Controlled clinical trials show that low GI diets can lower cholesterol [4], improve blood sugar control (HbA1c) and insulin sensitivity in diabetics [5], delay the return of hunger [6], and decrease body weight in adolescents [7,8].

The GI is defined as "the incremental area under the blood glucose response curve of a 50 g carbohydrate portion of a test food expressed as a percent of the response to the same amount of carbohydrate from a standard food taken by the same subject". The GI standardizes the glycemic response and accounts for between subject variability by averaging the results of testing at least 10 persons. The GI has been shown to be reliable in mixed meal testing environments demonstrating that the inclusion of fat or protein in a meal does not preclude the measurement of the GI of the carbohydrate content of that meal [9-12]. Foods have inherent GI values but there are several methods for effectively lowering the GI of a particular food. The addition of resistant starches or fiber products (psyllium, blackgram fiber, barley, oat beta-glucan) to the food may lower the GI [13-19].

Alpha-amylase, secreted in the saliva and by the pancreas, is responsible for breaking down starches into sugars that are consequently absorbed in the small intestine. Since the GI is a function of the rate of absorption of glucose in the gut, inhibition of alpha-amylase may result in a lowering of the GI. A partially purified white bean product has been shown to decrease post-prandial increases in plasma glucose [20,21].

Phase 2 is a dietary supplement derived from the common white kidney bean (*Phaseolus vulgaris*) that has been shown to inhibit the digestive enzyme alpha-amylase in vitro [22]. The objective of this study was to determine whether Phase 2 could lower the effective GI of a common high glycemic food product. We hypothesized that addition of the Phase 2 to white bead would affect the GI of the white bread.

## Methods

The Phase 2 product is a water extract of the white kidney bean (Phaseolus vulgaris) standardized to alpha-amylase (8;12;15;39) inhibiting units (Pharmachem Laboratories, Kearny, NJ). Phase 2 is produced from non-GMO whole white kidney beans, which are ground and then extracted for 4 hours. The liquid is filtered and concentrated under vacuum. The extract is filtered again, and then pasteurized before being spray dried. The product was dosed as powder (mixed in butter) and in capsule form. Phase 2 is odorless and tasteless. Wonder brand white bread (Interstate Bakeries, Kansas City, MO), which was purchased at one time, was utilized in this study.

### Subjects and Study Design

Fifteen healthy volunteer subjects between the ages of 24 and 44 and a BMI between 18 and 25 (kg/m^2^) were screened at the Medicus Research facility in Northridge, CA. IRB approval was obtained from the Copernicus Group IRB (Cary, NC) prior to any study related procedures. Good Clinical Practice (GCP)'s were followed throughout the study. All subjects gave informed consent according to GCP guidelines prior to initiating any study procedures. Screening fasting glucose levels were ≤ 100 mg/dL. Subjects with any active eating disorders, gastrointestinal illness or history of gastrointestinal surgery, diabetes or other endocrinologic disorders were excluded. Subjects underwent a history and physical examination by a board certified physician. All women of child bearing potential were given a urine pregnancy test and required to use appropriate methods of contraception during the active study. In order to standardize the glycemic response on the each study test day, subjects were required to consume only a diet of standardized prepared low-fiber frozen foods [23] containing a minimum of 100 g of carbohydrates. The purpose of the low-fiber diet is to minimize the potential residual blood sugar effects of slowly digested and absorbed complex carbohydrates which may be present up to 1 day after consuming them. Subjects were also required to fast for 10 hours prior to their study visit.

GI testing with 10 subjects was completed according to the FAO/World Health Organization (WHO) guidelines with standard methodology using glucose as the standard food [24]. During the standardization phase of the study, subjects reported to the study center 3 times during which they received 50 g net carbohydrates in the form of glucose. At each visit subjects had their capillary blood glucose measured 9 times over 2 hours. Capillary blood collections and multiple GI measurements were performed during the two hour interval as the recommended technique to reduce the measurement errors [25].

During the active phase of the study, subjects reported to the study center 7 times during which they received 50 g net carbohydrates in the form of white bread with butter either by itself or with a form of Phase 2. The serving of bread used to obtain 50 g of net carbohydrates was determined from the package label information. Butter was obtained in standardized plastic "pats" and each serving was 5 g, 36 kcal and contained 0 carbohydrates. The amount of butter was standardized for each test dose so that each subject received the same amount of butter at each visit regardless of how much test product they received. Although fat may affect the GI of foods [26], there was consistency in the study in that it was included in both the control and test groups. The test product was given at dosages of 1500 mg, 2000 mg, and 3000 mg in capsule form and 1500 mg, 2000 mg, and 3000 mg in powder form. The powder form of the test product was mixed into the butter which was spread on the bread. The capsules were taken immediately prior to the ingestion of food. During each visit subjects again had their capillary blood glucose measured 9 times over 2 hours.

The white bread was consumed within 5 minutes after which subjects remained in a semi-recumbent position throughout the duration of the study visit (unless they need to use the restroom) to reduce variability in oro-cecal transit time [27]. The only beverage allowed during the testing session was ice water. The test meals were administered in a random order and the test visits were less than 2 weeks apart.

### Analyses

Capillary blood was analyzed for blood glucose using the Ascensia Contour glucometer (Bayer Healthcare, Mishawaka, IN). Blood was drawn twice at baseline and then at times 0 (start of meal), 15 min, 30 min, 45 min, 60 min, 90 min and 120 min.

### Questionnaires

10 point Likert scales for diarrhea, flatulence, abdominal bloating, abdominal cramping, nausea, boborygmi (bowel sounds), and soft stools were filled out hourly and at the end of each test period.

### GI Calculation

The GI was calculated according to the FAO/WHO standard [24], which utilizes capillary blood glucose measurements. As per this protocol, when a blood glucose value fell below the baseline, only the area above the fasting level was included.

### Statistical Analysis

Statistical analysis was performed using t-tests to compare the mean differences within groups and between the various formulations/doses and the white bread control.

## Results

### Subjects

Fifteen (15) subjects were screened and 13 subjects (38% men and 62% women) completed this 6-arm open-label controlled crossover trial. Two subjects were withdrawn because their blood glucose went above 200 mg/dL in the glucose tolerance test during the standardization phase. These individuals were excluded as they did not have a "normal" glycemic response and may have been insulin resistant.

### Impact on GI

There was the appearance of a dose related effect of Phase 2 on the GI response to white bread with both the powder and capsule formulations (Table 1). For the capsule formulation, the 1500 mg dose had no effect on the GI and both the 2000 mg and 3000 mg capsule doses caused insignificant reductions in GI. For the powder, the 1500 mg and 2000 mg doses caused insignificant reductions in the GI, and the 3000 mg dose had a significant effect (-20.23 or 34.11%, p = 0.023)

**Table 1:** Phase 2 impact on GI

**Formulation**	**GI**	**% Change from white bread control**	**P Value**
Control	59.3 ± 24.7		
1500 mg Capsule	61.9 ± 2.6	-4.39	0.77
2000 mg Capsule	45.1 ± 14.2	24.01	0.076
3000 mg Capsule	46.8 ± 12.5	21.05	0.11
1500 mg Powder	43.6 ± 15.7	26.41	0.11
2000 mg Powder	45.2 ± 14.1	23.76	0.16
3000 mg Powder	39.1 ± 20.2	34.11	**0.023**

The GI of white bread control compared to the GI of white bread plus different doses and formulations of Phase 2. GI values are presented as mean ± SD, n = 13. P values are the result of comparisons between the control and treatment values.

### Safety

All of the dosages and formulations appeared to be well tolerated as there were no differences in the responses to the 10 point Likert scale for gastric effects. No adverse events were observed or reported during the study.

## Discussion

The data from this study demonstrates decreases in the GI of white bread with Phase 2 in both capsule and powder formulations. The decrease was statistically significant with the powder form at 3000 mg. The data suggest a possible dose dependent effect with a preference for the powder form. The lesser effect with the capsule formulation may reflect a reduced bioavailability of the white bean extract, perhaps due to the time required for capsule dissolution.

A limitation of the study was the lack of blinding of participants and study staff. Another limitation of the study is its small sample size, which was based upon the FAO/WHO guidelines for GI testing. Several methodologies were employed to diminish the inter and intra-subject variability inherent in GI testing including the use of glucose rather than white bread during the standardization phase, standardization of meals the day prior to each visit, restricting the inclusion criteria to certain age and BMI criterion, and the semi-recumbent position during the study to standardize oro-cecal transit time. An inherent person-to-person variability in these results is expected and GI calculation as an average takes these factors into account [28].

Previous pilot studies have been conducted with Phase 2 indicating a potential lowering of post-prandial blood glucose levels [29]. A three arm crossover study performed on 20 subjects compared two doses of Phase 2 (515 mg and 750 mg in powder form mixed with food) to placebo [30]. The subjects were given a standardized meal containing 64 g of carbohydrates (including 6 g dietary fiber and 19 g sugars). Serial glucose levels were measured every 10 minutes for 60 minutes using the One Touch Ultra blood glucose monitoring system. The 750 mg Phase 2 group demonstrated significantly lower blood glucose levels at 10, 20, and 30 minutes (p < 0.01) compared to both the 515 mg dose and with the placebo. The 515 mg Phase 2 group showed significantly lower blood glucose at 10, 20 (p < 0.01) and at 30 minutes (p < 0.05) compared with placebo. The blood glucose area under the curve was lower in the 750 mg group compared with the two other groups, but did not reach statistical significance (p < 0.1).

These data from preliminary single meal studies suggest that alpha-amylase inhibitors might be effective in decreasing the absorption of glucose from a carbohydrate containing meal, or might increase the time period over which a single load of carbohydrates is digested. These changes form the basis for the hypothesized mechanism of action of alpha-amylase inhibitors in reducing the glycemic index.

The benefits of reducing the effective glycemic index of food are indicated for those with impaired glucose metabolism [29,31,32]. However not all clinical studies with diabetics have been positive [33,34] and a workshop on the subject concluded that dietary fiber may be an important additional, independent, factor in health outcomes [35].

The present results merit further study with a larger number of volunteers. In addition, it would be worthwhile to determine if 2000 mg and 3000 mg doses of powder and/or capsule forms of Phase 2 can reduce the GI's of other high GI foods such as pasta or rice.

## Conclusion

The GI of white bread was significantly decreased by the addition of 3000 mg of the Phase 2 brand white bean extract in powder form. Other dosages and formulations, with the exception of the 1500 mg capsule form, showed a trend towards a reduction in GI. With the appropriate dose and formulation, the Phase 2 white bean extract may be a novel and potentially effective method for reducing the GI of existing foods without modifying their ingredient profile. Given the interest and potential benefits of low GI diets, further studies of Phase 2 with a larger study population and in combination with other high GI foods are indicated.

## Competing interests

Medicus Research has ongoing research support grants from Pharmachem Laboratories. Dr. Udani has provided consulting services to Pharmachem Laboratories. The authors and Medicus Research do not endorse any brand or product.

## Authors' contributions

JKU was the Principal Investigator. BBS did the analysis. JKU, BBS, MLB and HGP contributed to writing the manuscript.
